# Fusion expression of Occludin extracellular loops and an α-helical bundle: A new research model for tight junction

**DOI:** 10.1371/journal.pone.0175516

**Published:** 2017-04-27

**Authors:** Xiaojing Chi, Xia Zhao, Wei Wang, Yuqiang Niu, Min Cheng, Xiuying Liu, Sheng Cui, Wei Yang

**Affiliations:** MOH Key Laboratory of Systems Biology of Pathogens, Institute of Pathogen Biology, Chinese Academy of Medical Sciences, Beijing, China; SRI International, UNITED STATES

## Abstract

Tight junctions (TJs) are the outermost structures of intercellular junctions and are highly specialized membrane domains involved in many important cellular processes. However, most TJ proteins are four-time transmembrane proteins and are difficult to express in their correct soluble form, which limits their functional study and therapeutic application. Human occludin (OCLN) is a major component of TJs and an essential co-receptor for hepatitis C virus (HCV) cell entry. To explore expression strategy for recombinant TJ proteins possessing integrated and functional extracellular loops, OCLN was here used as a model molecule, and several prokaryotic fusion constructs were designed by docking OCLN extracellular loops (ECLs) to HIV-1 gp41 NHR and CHR six-helical bundle (6HV1); then their biophysical features and anti-HCV activity were evaluated. The proteins were successfully expressed and purified in *E*. *coli*, and the double-loop constructs (D1ECL1S+D2ECL2 as a representative) were found to have more potent HCV neutralizing activity than single-loop constructs at non-cytotoxic concentrations. Circular dichroism studies indicate that D1ECL1S+D2ECL2 adopt stable α-helical folds consistent with design. Thermal denaturation assay indicated that D1ECL1S+D2ECL2 is highly stable at 80°C (melting temperature, T_m_, of 89.08 ± 2.0°C) and comparable in stability to the 6HV1 scaffold. Moreover, the time-of-addition experiment revealed that D1ECL1S+D2ECL2 predominantly functioned during the early stages of HCV entry. Taken together, these findings provide a novel strategy for recombinant TJ protein expression *in vitro*, which may shed light on functional and structural studies for TJs and may provide a new avenue to drug development.

## Introduction

Tight junctions (TJs) are present at the apical ends of lateral membrane surfaces of epithelial and endothelial cells and form a series of discrete sites of apparent membrane fusion involving the outer leaflet of plasma membranes of adjacent cells. There are more than 30 known proteins involved in TJs, including transmembrane proteins such as occludin (OCLN), claudins, junctional adhesion molecules, and cytoplasmic adaptor molecules such as ZO-1, ZO-2 and ZO-3[1]. TJs play key roles in the barrier function and fence function in normal cells. TJ dysfunction is related to edema, jaundice, diarrhea, and blood-borne metastasis[2]. Recently, some TJ proteins have been reported to be cell receptors for viruses and pathogenic bacteria[3]. Although the close relationship between TJs and diseases is widely recognized, lack of efficient expression method of recombinant TJ proteins hindered intensive structural and functional studies for TJs. This is also an obstacle for targeting TJs during drug development.

OCLN is a 60-kDa integral membrane protein of TJ fibrils that spans the membrane four times with three cytoplasmic domains and two extracellular loops (ECLs). The first ECL has a high tyrosine and glycine composition, whereas the second ECL is rich in tyrosine residues[4]. Both ECLs consist solely of uncharged residues with the exception of one or two charged residues adjacent to the membrane. OCLN localizes to tight junctions, and its overexpression is known to increase transepithelial resistance in mammalian epithelial cells. It is also worth noting that OCLN is one of the major co-receptors for HCV infection[5].

HCV is an enveloped RNA virus in the Flaviviridae family that has a single-stranded, positive-sense RNA genome of 9.6 kilobases (kb)[6]. The first step of HCV infection is viral particles interaction with host membrane molecules including low-density lipoprotein receptor (LDLR)[7], scavenger receptor class B type 1 (SR-BI)[8], CD81[9], claudins (CLDNs)[10], occludin (OCLN)[5], Niemann-Pick C1-like 1 (NPC1L1), and transferrin receptor 1 (TfR1). Among these host entry factors, CD81 and OCLN are human-specific co-receptors, which restrict the natural host of HCV[11]. Though the function and crystal structure of human CD81 has been determined, the OCLN crystal structure is not available for the difficult expression[4].

The envelope glycoprotein of human immunodeficiency virus type 1 (HIV-1) is made of gp120 and gp41. Previous studies identified an α-helical domain within gp41 composed of a trimer of two interacting peptides. The crystal structure of this complex, composed of the peptides N36 and C34, is a six-helical bundle[12]. Three N36 helices form an interior, parallel coiled-coil trimer, while three C34 helices pack in an oblique, antiparallel manner into highly conserved, hydrophobic grooves on the surface of this trimer. The fusion peptide region, which is inserted into cellular membrane, is the main function domain in virus entry. In theory, similar structures could form when the fusion peptide region is replaced by other fusion or entry region, given the similarity in their proposed fusion mechanisms.

The goal of the current work was to design protein mimics of OCLN by docking to the α-helical bundle segment of HIV gp41, which could be used as model systems to study folding stability and potentially aid in development of inhibitors of HCV infection. To this end, model proteins consisting of the alternating NHR and CHR segments from HIV-1 gp41 linked by short, flexible peptide linkers or ECLs were prepared from OCLN. Biophysical characterization reveals that these proteins adopt stable α-helical structures consistent with their design. Further analysis provided interesting insights into factors that the protein, named D1ECL1S+D2ECL2, can be a novel anti-HCV agent that blocks at early step of the entry process by targeting HCV particles. Importantly, these findings provide a novel strategy for TJ protein expression in vitro, which may improve the study of the function of TJs.

## Materials and methods

### Cells and reagents

Human hepatocyte Huh7.5.1 was provided by Dr. Francis Chisari (Scripps Research Institute). The HCV genotype 1b replicon-containing cell line (2−3+) was provided by Dr. Stanley Lemon (University of Texas Medical Branch, Galveston, TX, USA). HEK293T cells were obtained from ATCC. All cell lines were maintained in DMEM supplemented with Penicillin and Streptomycin, 1% NEAA, and 10% fetal bovine serum (Gibco, Carlsbad, CA, USA). Antibodies were obtained from Thermo Scientific (HCV Core) and Sigma (β-actin). Secondary antibodies are purchased from Santa Cruz (Santa Cruz, CA, USA), Jackson ImmunoResearch Laboratories (West Grove, PA, USA). Heparin, Isopropyl β-D-1-thiogalactopyranoside(IPTG) and were purchased from Sigma (St Louis, MO, USA). PEG-IFNα2b was from Schering Plough (Kenilworth, NJ, USA).

### Production of pseudoviral particles and cell culture-grown HCV (HCVcc)

Production procedure of HCVcc expressing firefly luciferase and HCV pseudoviral particles (HCVpp) was described elsewhere[13].

### HCVpp infection assay

To conduct the infection assay, Huh7.5.1 were seeded in a 96-well plate at the density of 2 ×104/well the day before infection. The next day, 100 μl of supernatant containing HCVpp, VSV-Gpp or bald virus was added into each well in the presence of 8 μg/ml of polybrene and 1 μl of 2 M HEPES (pH 7.55) and spin infected for 1.5 h in a table-top centrifuge (2500 rpm, 30°C), and followed by another 1.5 h incubation in a CO2 cell incubator. Cells were lysed at 48 h post spin-infection and assayed with Luciferase Assay System (Promega, Madison, WI, U.S.) in a Modulus Microplate Luminometer (Turner BioSystems, Sunnyvale, CA, U.S.). All experiments were performed in triplicate. Counts ranging from 10,000–900,000 were typical, whereas the background signal from bald virus infected sample was usually below 100.

### Cytotoxicity assay

Huh7.5.1 cells (105 per well) were treated with D1ECL1S+D2ECL2 or 6HV1 for the indicated concentration and period of time. After removal of D1ECL1S+D2ECL2 or 6HV1, cells were incubated for an additional 48 hours in 96-well plates. Cytotoxicity was determined using the MTT Cell Viability Assay Kit from R&D Systems (Minneapolis, MN, USA Cat# 4890-025-K).

### In-Cell Western (ICW)

Huh7.5.1 cells were seeded in 96-well plate format at 1×104 per well one day before HCVcc infection (MOI = 0.01). Twenty-four hours post virus infection, the cells were treated with proteins or 6HV1 for indicated time. Seventy-two hours post infection, the cells were fixed in the original wells with paraformaldehyde and permeablized with Triton X-100, followed with immunostaining with anti-HCV core antibody (1:400 dilution) and IRDye Secondary Antibody (1:1000 dilution, Li-Cor, Nebraska, USA). Images were obtained on Odyssey Infrared Imaging System (Li-Cor, Lincoln, NE, USA).

### HCVcc binding assay

HCVcc supplemented with 20 mM HEPES were added to Huh7.5.1 cells, seeded in triplicate in 24-well plates (4×104 cells/well), in the presence or absence of specified inhibitors for 1–3 h at 4°C with gentle rocking (MOI ≈1). The resulting cells were washed three times with cold phosphate buffered saline (DPBS), followed by total RNA isolation with TRIzol reagent (Invitrogen, Carlsbad, CA, USA). Quantification of RNA was conducted using QuantiFast SYBR Green RT-PCR Kit (Qiagen, Germantown, MD, U.S.) with an in-house developed protocol on a Step One Real-Time PCR system (Applied Biosystems). Primer sequences for the HCV RNA genome were forward: 5′-GCC TAG CCA TGG CGT TAG TA-3′ and reverse: 5′-CTC CCG GGG CAC TCG CAA GC-3′. The copy number of HCV RNA was calculated by comparing to a standard curve obtained with serial dilutions of a full-length HCV genome encoding plasmid.

### Confocal microscopy

Immunostaining of Huh7.5.1 cells was performed using antibodies against core as previously descriped[14,15]. Images were captured on a Leica SPW5 confocal microscope. Nucleus was stained with DAPI.

### Protein expression and purification

The gene sequence of 6HV1 was showed in the S1 File. The cDNA encoding N36 and C34 complex was stringed together by linkers (Linker1, linker3, linker5 sequence: SGGLEVLFQGPSGG; Linker2: GGS-GS-AAA-KL-GAS; Linker4: GSS-RS-GGG-VD-AAS). In this gene, there are two couples of Enzyme loci, BamHI/HindIII and BglII/SalI, the gene between these two sites could be replaced.The whole gene was cloned to pet28a plasmid, at NcoR and XhoI sites, expressing the N-terminal 6×His-tagged domain. Then the two extracellular loops of OCLN were insert into this gene. The expression and purification of different fusion protein followed essentially the same protocol. Briefly, plasmid was transformed to Rosetta (DE3) competent cells (Novagen, Madison, WI, USA).Bacterial cultures were grown in Luria-Bertani medium at 18°C. The induction was initiated by the addition of isopropyl -b-D- thiogalactop -yranoside to 0.5 mM when the culture reached an optical density at 600 nm of 0.4–0.6. The bacterial culture continued for overnight at 18°C after the induction. Bacterial cells were harvested by centrifugation at 6000 rpm for 20 min. Cell pellets were resuspended in a lysis buffer [50 mM Tris-HCl (pH 8.5) and 100 mM NaCl] and disrupted by ultrasonication. The soluble fraction of the lysate was discarded by centrifugation at 12,000rpm for 30 min. The purification of supernatant fusion proteins or inclusion bodies includes Ni-NTA(Qiagen, Germantown, MD, USA) affinity chromatography followed by thrombin digestion to remove the N-terminal 6× HIS tag. The protein was finally purified by size exclusion chromatography (Superdex 200 10/300 GL; GE Healthcare, Waukesha, WI, USA).

### CD spectroscopy

Final concentrations of D1ECL1S+D2ECL2 and 6HV1 were 10 μM in PBS. The CD spectra were acquired on a Jasco spectropolarimeter (modelJ-815; Tokyo, Japan) using a 1-nm band width with a 1-nm step resolution from 195 to 260 nm at room temperature. The spectra were corrected by subtraction of a blank corresponding to the solvent. Data were averaged over three accumulations. The α-helical content was calculated from the CD signal by dividing the mean residue ellipticity [θ] at 222 nm by the value expected for 100% helix formation (-33,000 * deg * cm2 * dmol 21). A thermal denaturation experiment was performed by monitoring the change in ellipticity [θ] at 222 nm at increasing temperature (20–98°C) using a temperature controller. The temperature was increased at a rate of 2°C/min; data were acquired using a 1-nm bandwidth at 222 nm at a frequency of 0.25 Hz. The melting curve was smoothened, and the midpoint of the thermal unfolding transition (Tm) values was taken as the maximum of the derivative d[θ]222/dT.

## Results

### Design of recombinant OCLN mimics by docking ECLs to a HIV-1 derived α-helical bundle

**Fig 1:**
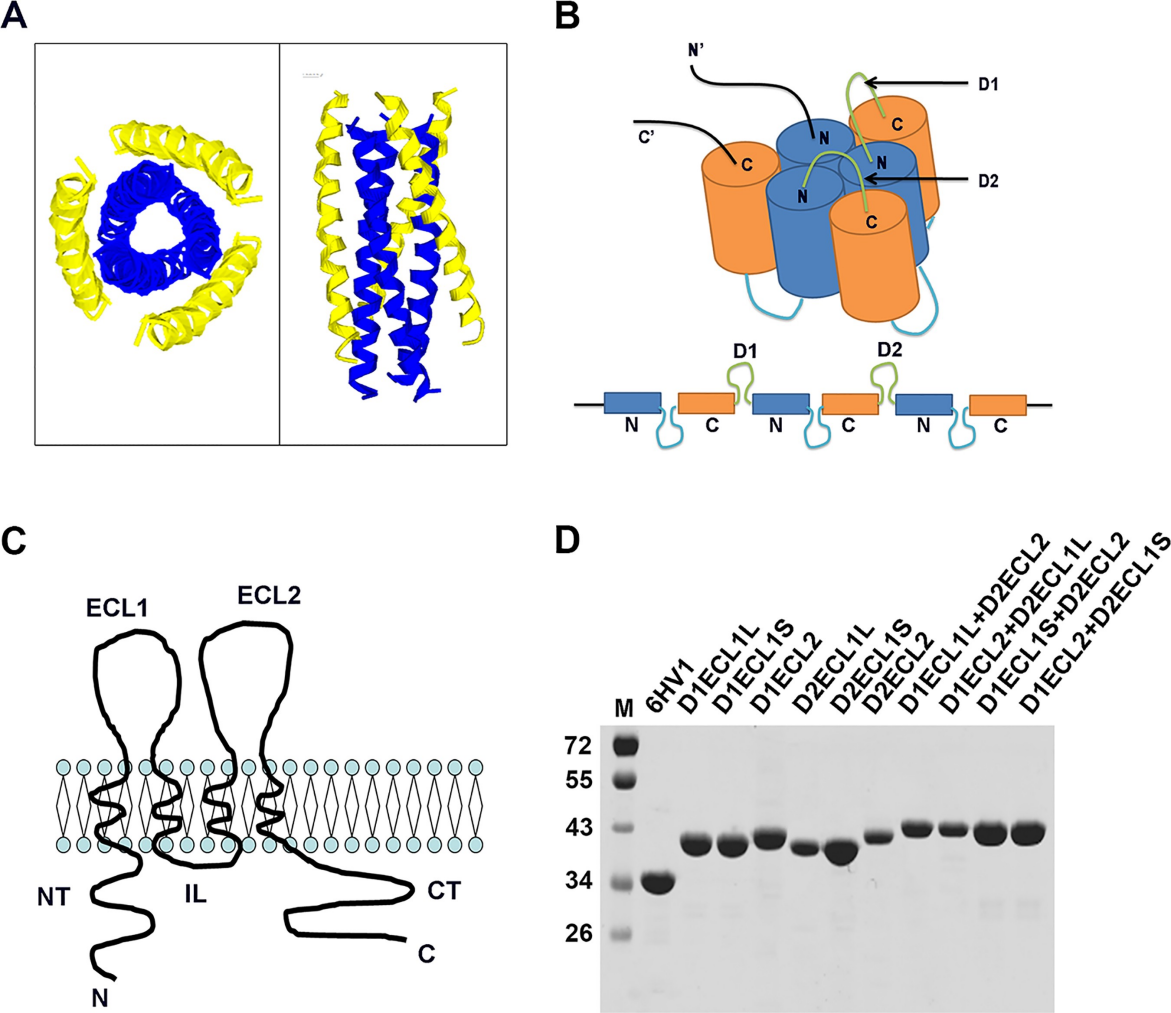
Design and expression of OCLN ECLs/6HV1 fusion proteins. **(A)** Overall views of the N36/C34 Complex (PDB ID 1AIK). The NHR is shown in blue and the CHR is shown in yellow. **(B)** Topology display of the six-helix bundle of 6HV1. D1 and D2 are the docking sites that can be replaced with OCLN ECLs. **(C)** Schematic structure of OCLN in plasma membrane. OCLN has four transmembrane domains (TM1–TM4), two ECL domains (ECL1, ECL2), one intracellular loop domain (IL), and both N-terminal (NT) and C-terminal (CT) domains. **(D)** SDS-PAGE analysis of the refolded and purified fusion proteins. In all cases, a single band was observed of expected molecular weight.

As with other TJ proteins, acquiring soluble OCLN full-length protein is still stumbling. Evidence from the current and other research groups showed difficulties in the expression or re-folding of both full-length and ECLs of OCLN in prokaryotic system. The evidence for the extended intermediate in HIV-1 gp41, called 6HV1, was obtained from designed peptides and proteins that bound the extended intermediate and prevented rearrangement to the six-helix bundle[16,17]. Proteins, peptides, and small molecules that bind protein mimics of the HIV-1 gp41 extended intermediate have been found to have antiviral activity and have been exploited for design of viral entry inhibitors[18,19,20,21]. For this reason, an expression strategy was designed to simulate natural OCLN protein by docking single or double ECLs to 6HV1. As shown in Fig 1A, the structure of N36/C34 complex is a six-stranded helical bundle[12]. Linkers were used to string together the N36 and C34 (Fig 1B). This tandem protein was used to simulate the transmembrane domains of OCLN to ensure correct conformation and the domain 1 (D1) and domain 2 (D2) can be replaced by other protein domains without influence the structure. OCLN is a tetraspanin protein with two ECLs (predicted online http://www.cbs.dtu.dk/services/TMHMM-2.0/) (Fig 1C), ECL1, and ECL2. ECL1 is predicted to have two forms, called ECL1L (long) and ECL1S (short), under different algorithms, whereas ECL2 has only one form(S1 Fig). In these designed proteins, 6HV1 D1 and D2 were replaced with OCLN ECL1S, ECL1L, or ECL2 (Table 1). To foster a high level of expression, the coding sequences were optimized based on *E*. *coli* codon bias. The fusion proteins containing hexahistidine tags were expressed in *E*. *coli*, purified from inclusion bodies, and then subjected to refolding conditions. All proteins were obtained in good yield and purity (Fig 1D).

**Table 1:** Combinations of the fusion protein constructs.

6HV1	D1	D2	MW(KD)
	ECL1L	—	37.7
	ECL1S	—	37.4
One loop insertion	ECL2	—	38.0
	—	ECL1L	37.7
	—	ECL1S	37.4
	—	ECL2	38.0
	ECL1L	ECL2	42.8
Two loops insertion	ECL1S	ECL2	42.5
	ECL2	ECL1L	42.8
	ECL2	ECL1S	42.5

### Anti-HCV effects of the fusion proteins

**Fig 2:**
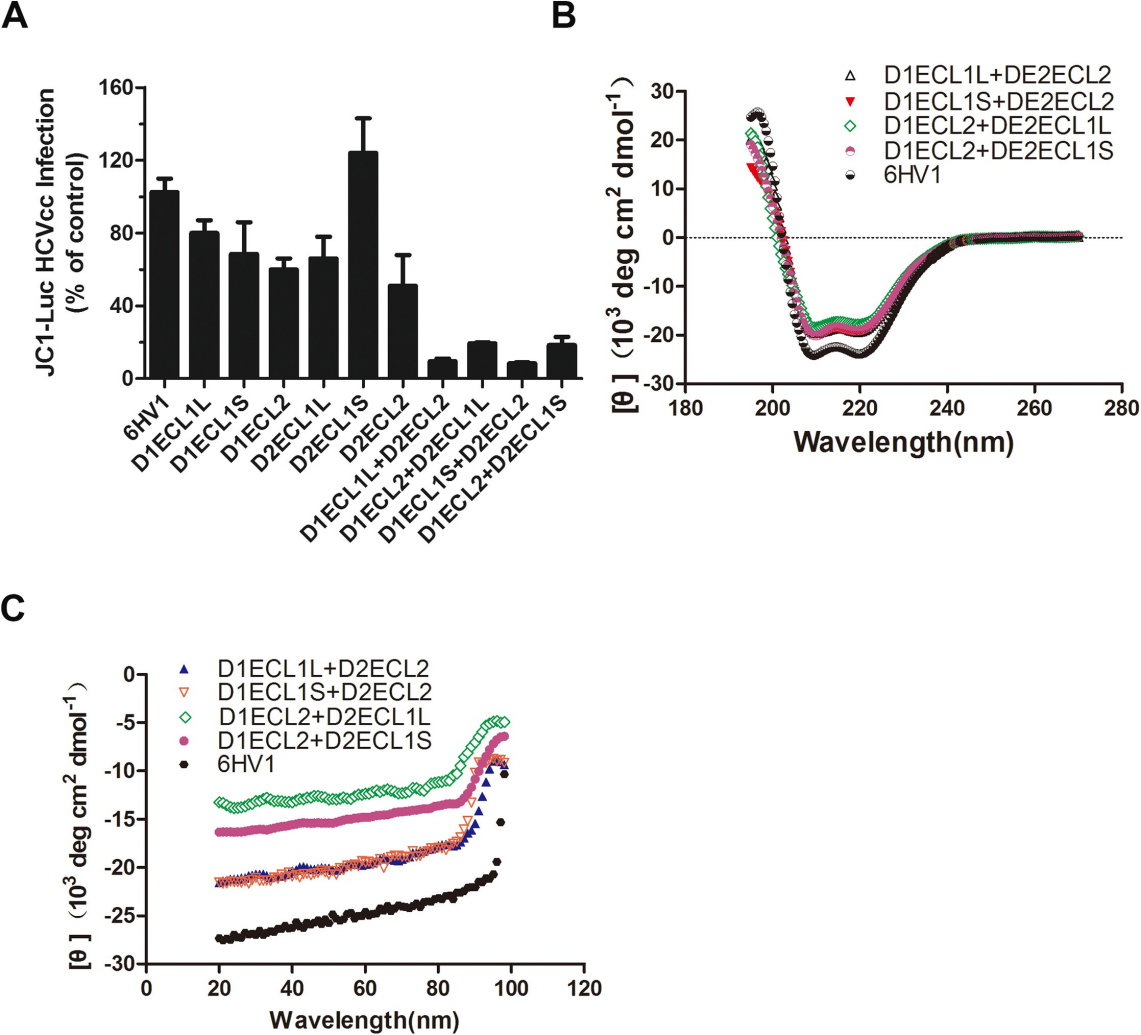
Anti-HCV effects of the fusion proteins and biophysical characterization of D1ECL1S+D2ECL2. **(A)** Effects of the fusion protein on HCVcc infection. Jc1-Luc HCVcc (MOI = 0.1) was used in the initial screening. Fusion proteins (1 μM) were premixed with the Jc1-Luc HCVcc and then added into Huh7.5.1 cells. 6HV1 scaffold protein was included as a negative control. Cells were lysed for luciferase activity measurement at 48 h after inoculation. Results are calculated as relative inhibition to counts obtained from 6HV1-treated cells (set to 100%). **(B)** CD spectra of D1ECL1S+D2ECL2, D1ECL1L+D2ECL2, D1ECL2+D2ECL1S, D1ECL2+D2ECL1L and 6HV1 in 20 mM sodium acetate. **(C)** Thermal denaturation of D1ECL1S+D2ECL2, D1ECL1L+D2ECL2, D1ECL2+D2ECL1S, D1ECL2+D2ECL1L and 6HV1.

Human OCLN is one of the major cell entry co-receptors of HCV and has been shown to be associated with HCV envelop glycoproteins[22]. The simulated fusion proteins with correct function should bind to the surface of incoming virions, and then competitively block the association between virus and cellular receptors and result in entry inhibition. A Jc1-Luc HCVcc system and Huh7.5.1 cells were used to evaluate the antiviral activity of the fusion proteins. As show in Fig 2A ECL1, single replacement had almost no or merely marginal inhibitory effects on HCV infection, whereas ECL2 single replacement showed a 40–50% inhibition, which is consistent with the fact that OCLN ECL2 is the major loop for HCV entry. Consistent with the current hypothesis, double ECLs replacement demonstrated a striking contrast from single ECL replacement and showed 80–90% inhibition. The fusion protein D1ECL1S+D2ECL2 was chosen as an example in subsequent experiments because of its extremely pronounced anti-HCV potency. For comparison, the 6HV1 scaffold protein failed to inhibit HCV inhibition (Fig 2A). This suggests that the current strategy for constructing a tetraspanin TJ recombinant protein by docking its ECLs to an α-helical bundle can mimic its membrane-bound activity to the greatest extent.

### Structural analysis of D1ECL1S+D2ECL2

gp41 is a six-helical bundle with six α-helical secondary structures. However, it is not clear whether the secondary structure of the fusion protein will change if other non-α-helical secondary structure domains are inserted into it. To test this hypothesis, CD spectroscopy of D1ECL1S+D2ECL2 was performed in a near physiologic solution. As shown in Fig 2B the CD spectra of D1ECL1S+D2ECL2 and 6HV1 are both displayed signatures of double minima at 208 and 222 nm, indicating the predominantly helical conformation in solution. D1ECL1S+D2ECL2 has less obvious α-helicity than 6HV1, which means that the two ECLs might not adopt α-helical structures. The thermostability of the fusion protein or scaffold control protein was determined by defining the midpoint of the thermal unfolding transition (T_m_) value. As shown in Fig 2C the insertion of two ECLs changed the stably folded of 6HV1 in solution, as evidenced by the decreased T_m_.

### D1ECL1S+D2ECL2 and HCV infection

**Fig 3:**
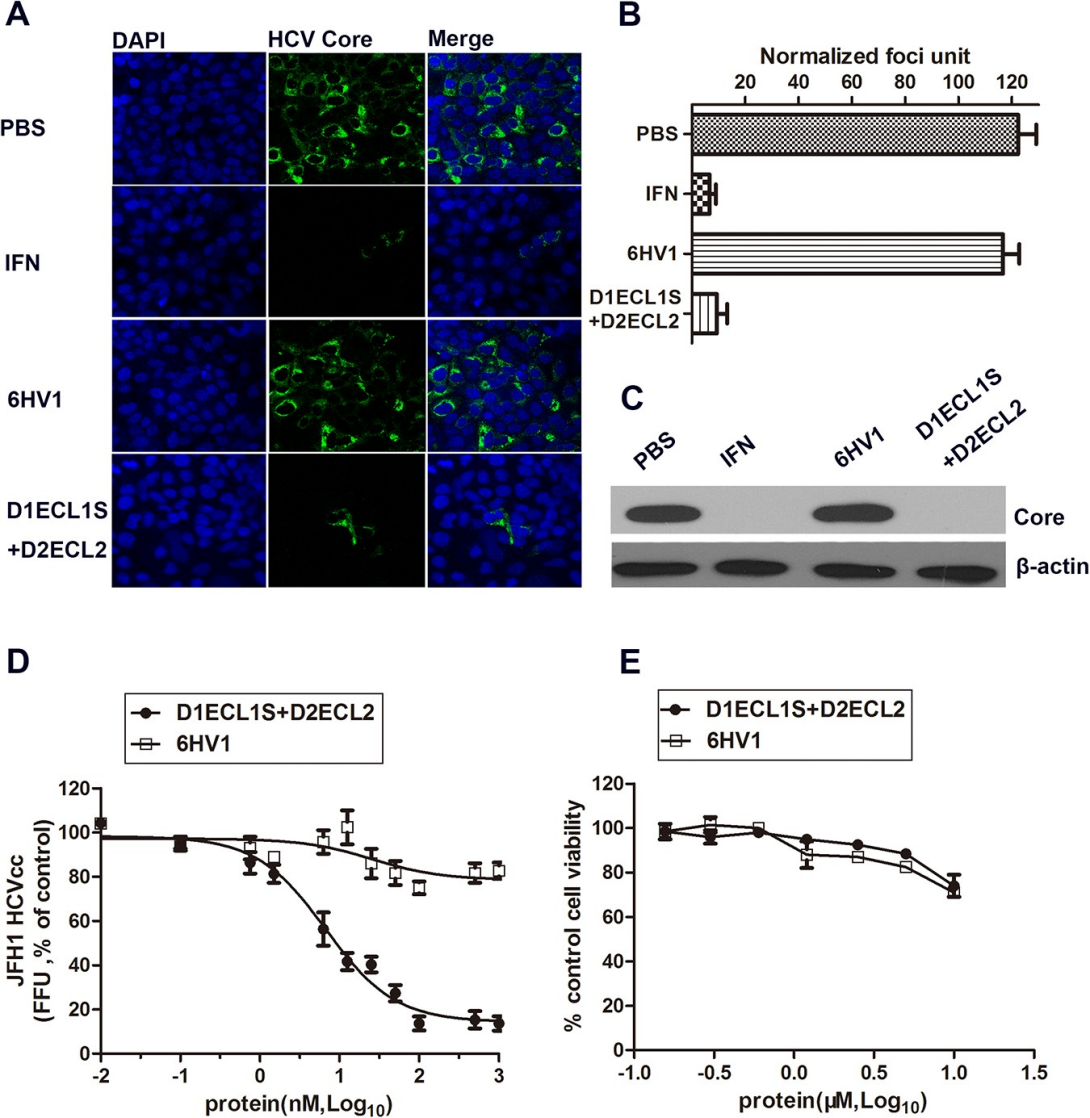
Determination of the anti-HCV activity and cytotoxic effect of D1ECL1S+D2ECL2. **(A)** JFH-1 HCVcc was premixed with PBS, IFN-α2b (100 U/mL), 6HV1 (100 nM) and D1ECL1S+D2ECL2 (100 nM), respectively, and then used to infect Huh7.5.1 cells. At 48 h post-infection, immunofluorescent staining was performed with antibody against HCV Core (green), and DAPI for nuclei (blue). **(B)** The number of HCV infected cells. **(C)** Same experimental design for treatments and infections was performed as described in section (A), and HCV core protein was measured by Western blotting. **(D)** Serially diluted D1ECL1S+D2ECL2 was mixed with JFH-1 HCVcc (MOI = 0.1) and then incubated with Huh7.5.1 cells for 12 h before media change. Foci formation unit (FFU) was counted at 48 hours after infection by staining cells with antibody against HCV Core. 6HV1 was included as a negative control. **(E)** Cytotoxicity of D1ECL1S+D2ECL2 and 6HV1 for Huh7.5.1 cells were determined by the MTT assay.

Soluble receptors may be developed for efficient antivirals. In this way, to further understand the antiviral potency and possible cytotoxicity of D1ECL1S+D2ECL2 fusion protein, we assessed these features using hepatocyte Huh7.5.1 infection model with HCV genotype 2a isolate from a patient with fulminant hepatitis in Japan (JFH-1 HCVcc). Results showed that D1ECL1S+D2ECL2 treatment significantly inhibited HCV infection in Huh7.5.1 cells with a comparative efficiency to interferon (IFN) treatment. However, the 6HV1 scaffold, which served as a negative control, failed to show any suppressive effects on HCV (Fig 3A, 3B and 3C). Furthermore, serial dilutions of the fusion protein were performed and IC_50_ values was calculated from the concentration response curves (IC_50_ = 9.71±4.97 nM, Fig 3D). To rule out any confounding effect due to cytotoxicity, the 50% cytotoxic concentration of D1ECL1S+D2ECL2 was also determined and the therapeutic index was estimated to be more than 100 (Fig 3E). These results demonstrate that the fusion protein D1ECL1S+D2ECL2 has the biological activity, and appears to be a HCV infection inhibitor with high potency.

### D1ECL1S+D2ECL2 inhibits HCV entry

**Fig 4:**
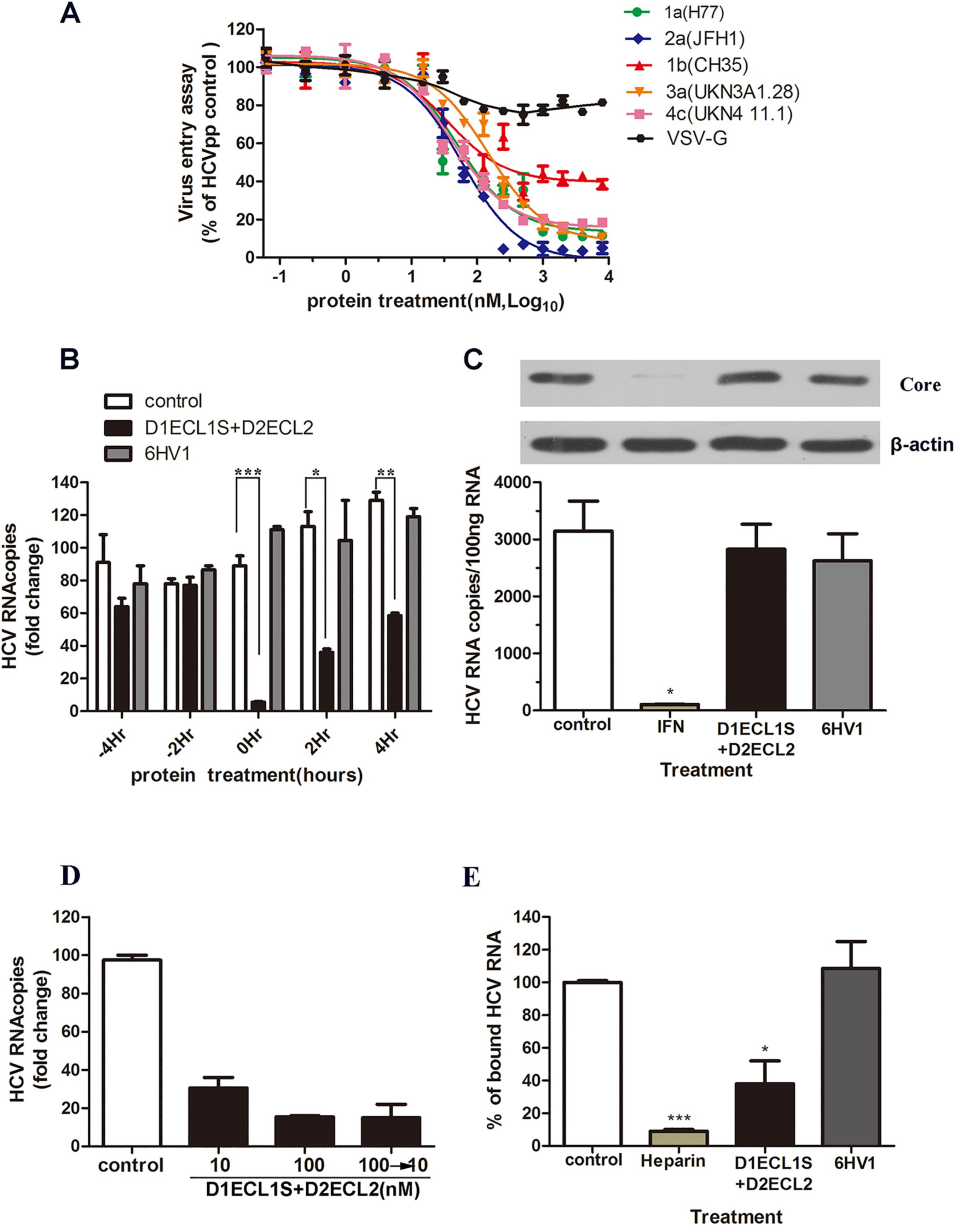
Antiviral mechanism of D1ECL1S+D2ECL2. **(A)** VSV-Gpp (VSV-G-pseudotyped lentivirus) and HCVpp were packaged with E1E2 from major genotypes were used to infect Huh7.5.1 cells in the presence of serially diluted D1ECL1S+D2ECL2 for an entry assay. The results were calculated relative to the phosphate-buffered saline treated cells. **(B)** D1ECL1S+D2ECL2, 6HV1 or phosphate-buffered saline was added to Huh7.5.1 cells 2 or 4 h prior to (-2 h and -4 h), after (+2 h and +4 h) inoculation of JFH-1 HCVcc (MOI = 0.1), or together with the virus (0 h). For the -2 and -4 h cells, proteins were washed away before viral inoculation, whereas the 0, +2, and +4 h cells were incubated with protein along with the virus for a total of 12 h before the change in media. At 48 h after infection, the intracellular HCV RNA was quantified by real-time qRT-PCR. Results were calculated as fold of change over the value obtained from phosphate-buffered saline 0 h cells (set to 100–10). ***P<0.001,** *P* <0.01,*P<0.05. **(C)** 2^−^3^+^ HCV replicon cells were treated with phosphate-buffered saline, Interferon (IFN, 100 U/mL), D1ECL1S+D2ECL2 or 6HV1 for 48 h. The core protein level was measured by Western blotting (upper), and the intracellular HCV RNA was determined by qRT-PCR (lower). The experiment was performed in triplicate, and the error bars represent the standard error of the means (SEM). **(D)** HCVcc were pretreated with 100 μM D1ECL1S+D2ECL2 prior to inoculation and used to infect cells after 10-fold dilution of the inoculums (100–10). Infection with untreated virus was performed in parallel in the presence of the D1ECL1S+D2ECL2 concentrations indicated. **(E)** HCVcc was mixed with phosphate-buffered saline, D1ECL1S+D2ECL2, 6HV1, or heparin (200 μg/mL) and then added to precooled Huh7.5.1 cells and allowed for 3 hours incubation at 4°C. Cells were then thoroughly washed in phosphate-buffered saline and subjected to RNA isolation. Cell surface bound HCV was quantified by qRT-PCR. ***P<0.001,*P<0.05.

As a protein from HCV co-receptor, it is most likely that D1ECL1S+D2ECL2 inhibits HCV infection at the entry step. To test this hypothesis, the antiviral activity of D1ECL1S+D2ECL2 was first determined using HCVpp, which was prepared using HCV E1E2 from genotypes 1–4. The IC_50_ of D1ECL1S+D2ECL2 ranged from 35.93 nM to 145.7 nM depending on the genotypic origin of the envelope proteins (Fig 4A). However, D1ECL1S+D2ECL2 did not inhibit the entry of VSV-G-pseudotyped lentivirus, suggesting the specificity of inhibition (Fig 4A). Then, the fusion protein was added to the Huh7.5.1 cells 4 h or 2 h prior to (-2 h and -4 h), after (+2 h and +4 h) inoculation of HCVcc, or together with virus (0 h). It was observed that D1ECL1S+D2ECL2 exhibits the best inhibitory activity when added to the cells together with the virus and less effective at +2 h and +4 h, but no inhibition was observed when the protein was added before infection (Fig 4B). To eliminate the influence of D1ECL1S+D2ECL2 on HCV replication step, a full-length genotype 1a HCV replicon model was used. 2^−^3^+^ replicon cells were treated with D1ECL1S+D2ECL2 (100 nM), IFN (0.5 nM) or DMSO for 24 h. The viral genomic RNA and Core protein were detected using qRT-PCR and Western blotting. Consistent with our expectation, D1ECL1S+D2ECL2 treatment had no impact on HCV RNA replication or the intracellular HCV Core protein level in replicon cells (Fig 4C). Taken together, D1ECL1S+D2ECL2 appears to be a highly potent inhibitor of HCV entry.

### D1ECL1S+D2ECL2 impedes HCV cell entry by directly acting on the virus

Next the mechanism whereby D1ECL1S+D2ECL2 inhibits HCV entry was determined. In theory, as the OCLN functional domain, D1ECL1S+D2ECL2 can inhibit HCV entry by acting directly on HCV particles. To test this possibility, HCV was pre-incubated with D1ECL1S+D2ECL2 (100 nM) for 1 h before inoculation, and then the mixture was diluted 10 times before adding to the cells (at a final concentration of 10 nM). Importantly, the MOI was kept constant under all of the conditions. As shown in Fig 4E, the inhibitory effect of D1ECL1S+D2ECL2 was more pronounced when the virus was pre-incubated with the protein prior to inoculation, demonstrating that D1ECL1S+D2ECL2 acted directly on the HCV particle. At the same time, a virion binding assay was performed at 4°C. A well characterized HCV-cell binding inhibitor, Heparin, was used as a positive control[23]. HCV virions pre-incubated with PBS, D1ECL1S+D2DECL2, heparin or 6HV1 were allowed to bind to Huh7.5.1 monolayers at 4°C. Unbound virions were washed away and the bound virions were determined by quantifying HCV genomic RNA. HCV binding to target cells is expressed as a percentage relative to the PBS vehicle-treated control. As shown in Fig 4F, D1ECL1S+D2ECL2 decreased the amount of HCV bound to the target cells. In summary, D1ECL1S+D2ECL2 appears to directly impair the ability of the virus itself to enter the target cells.

## Discussion

TJ tetraspanin protein ECLs are responsible for TJ formation, signal sensing and even pathogen invasion as the cellular co-receptors. In this study, we demonstrated a novel strategy for expressing tetraspanin TJ proteins by fusing ECLs to an HIV-1 gp41 glycoprotein-derived α-helical bundle scaffold. This method allows expression of either single loop or double loops of human OCLN. The recombinant fusion proteins were normally enriched in the inclusion body but easily refolded and purified. Using HCV neutralizing assay, results showed that the double-loop constructs possessed significantly more biological activities than the single-loop constructs, suggesting that the structures of the fusion proteins are comparable to those of their native homologous proteins and both extracellular loops need to work synergistically. A model was established for multiple transmembrane proteins whose full-length proteins are difficult to express, providing an excellent tool for functional and even structural studies.

In recent years, an increasing amount of evidence has suggested the importance of TJs in the infection of several viruses, making it clear that studying the role of TJs during viral infection is important to achieving a better understanding of how viruses make use of the cellular machinery in order to complete their infection cycle. TJs are multiprotein complexes formed by different integral membrane proteins. The TJ-associated marvel proteins (TAMPs) are the important transmembrane component of the TJs. This family of proteins has three members, OCLN, tricellulin, and marvelD3. A lot of research has been conducted to determine the functions of OCLN. Like many other viruses, HCV can bind several different molecules on the host factors, including heparan sulfate proteoglycan, LDLR, SR-BI, CD81, CLDN1, and OCLN. The previous study has suggested that when OCLN is overexperessed in non-human cells, the uptake of the virus is highly increased. The step at which OCLN participates in HCV entry has yet to be determined, partially because the OCLN functional domains are not available for the difficult expression[24].

In the current study, OCLN was selected as a research object. Two ECLs from OCLN were expressed separately. Unfortunately, there was almost no soluble expression, and the inclusion body formed a misfolded structure without any function after a refolding step. Then the two ECLs were recombined into a six-helical bundle to mimic the HIV-1 envelope glycoprotein-gp41. These ECLs have been used as entry inhibitors and mimics of the extended intermediate. All the fusion proteins containing hexahistidine tags were obtained in good yield and purity.

An analogue of the natural OCLN, D1ECL1S+D2ECL2 has the strongest inhibitory activity. The inhibitory effects of double ECL insertion proteins were all better than single ones. This phenomenon suggested that though the second ECL is critical to HCV infection in hepatic cells[24], the first ECL may play an important role in the process by adjusting the conformation of the second ECL in HCV infection. Results also demonstrated that the OCLN fusion protein inhibits the entry of HCVcc as well as that of HCVpp, regardless of genotype, and that D1ECL1S+D2ECL2 acts early during entry by inhibiting association of the virus to the surface of the cell. These results show that D1ECL1S+D2ECL2 acts on HCV virions itself and that it is active against most of HCV genotypes.

Currently, the direct antiviral agents all target HCV non-structural proteins to inhibit its replication[25,26]. The development of inhibitors against other steps of the viral life cycle, including cell entry, can help expand the scope of antiviral strategies against hepatitis C. HCV receptors of HCV are the most important ring in the entry stage. This recombination of the ECLs of OCLN, which can efficiently inhibit HCV entry-related events, can serve as a candidate agent or potential lead for the development of HCV entry inhibitors.

Occludin was identified as the first integral membrane protein localized at tight junctions, it has a MARVEL domain, which have been shown to associate with cholesterol rich membrane domains and are thought to be involved in the biogenesis of endocytic vesicles and in the organization of specialized membrane domains like the TJs[27]. Although some TJs proteins, such as mCldn15, mCldn19, crystal structure are clear, it is hard to get active TJs proteins in prokaryotic expression system which lead to difficult to get insight into the architecture and characterization of these TJs. Our findings provide a novel strategy for TJ protein expression *in vitro*, which may shed light on better ways to study the function of TJs.

## Supporting information

S1 Fig The ECL1 and ECL2 amino acid sequence. (TIF)

S1 File The gene sequence of 6HV1. (DOC)
