# Supplementary figures and images for: Fusion expression of Occludin extracellular loops and an α-helical bundle: A new research model for tight junction
Source: PLoS One. 2017 Apr 27;12(4):e0175516. doi: 10.1371/journal.pone.0175516 (PMC5407606; doi:10.1371/journal.pone.0175516)

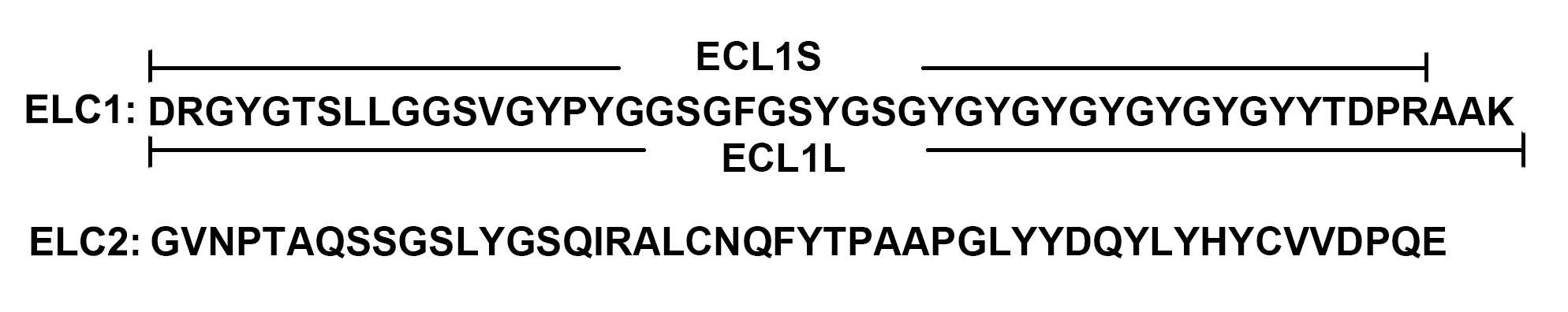

Supplement: S1 Fig — (TIF) [file pone.0175516.s001.tif]
